# Hydatid Cysts of Liver and Portal Hypertension

**DOI:** 10.1155/1990/54578

**Published:** 1990

**Authors:** Ali Emre, Orhan Arioğul, Aydin Alper, Attilâ Ökten, Ali Uras, Süleyman Yalçin

**Affiliations:** 1 Departments of Hepatobiliary Surgery and Hepatology Istanbul Medical Faculty Çapa Istanbul Turkey; 2 9-10 Mah A7B 95 Atakōy Istanbul 34750 Turkey

## Abstract

Two cases of portal hypertension due to hydatid cysts of the liver are reported. In one of the patients, symptoms were secondary to obstruction of inferior vena cava and hepatic outflow tract. The other patient was operated on with a diagnosis of extrahepatic presinusoidal portal hypertension caused by extrinsic compression of the liver by an hydatid cyst. Although hydatidosis is a benign disease, it can produce serious complications as in these reported cases. Therefore hydatidosis should be remembered amongst the causes of portal hypertension in countries where the disease is endemic.


*HPB Surgery* 1990, Vol. 2 pp. 129–133
Reprints available directly from the publisher
Photocopying permitted by license only

© 1990 Harwood Academic Publishers GmbH
Printed in the United Kingdom

### HYDATID CYSTS OF LIVER AND PORTAL HYPERTENSION

ALI EMRE,* ORHAN ARIOĞUL, AYDIN ALPER, ATTILÂ ÖKTEN, ALI URAS, AND SÜLEYMAN YALÇIN

*Departments of Hepatobiliary Surgery and Hepatology of Istanbul Medical Faculty, Çapa, Istanbul, Turkey.*

*(Received 17 January 1989)*

Two cases of portal hypertension due to hydatid cysts of the liver are reported. In one of the patients, symptoms were secondary to obstruction of inferior vena cava and hepatic outflow tract. The other patient was operated on with a diagnosis of extrahepatic presinusoidal portal hypertension caused by extrinsic compression of the liver by an hydatid cyst. Although hydatidosis is a benign disease, it can produce serious complications as in these reported cases. Therefore hydatidosis should be remembered amongst the causes of portal hypertension in countries where the disease is endemic.

KEY WORDS: Hydatid cyst, Budd-Chiari syndrome, portal hypertension

### INTRODUCTION

Although portal hypertension is mentioned as a complication of hydatid disease in classical textbooks, reported observations and numbers of cases are very few in comparison with others like rupture into the biliary tract, pleural and peritoneal spaces which have been thoroughly investigated and discussed in the literature[1,2]. In this short report, we present two rare cases of hydatid cyst complicated by portal hypertension.

### CASE REPORTS

Between the years 1977–1988, 203 cases of liver hydatid cysts were treated in our Unit. Complications encountered in this series are shown in Table 1.

*Case 1*: a 45 year-old female. She was admitted to the Hospital complaining of pain in the right upper quadrant and back, high fever and abdominal distention. She had been operated on twice, 16 years and 1 year previously because of liver hydatid cysts. On physical examination, ascites, caval and portal type collaterals on the abdominal wall and even on the back were observed (Figure 1). The liver, 12 cm below costal margin was blunt-edged and slightly painful upon pressure. Moreover, 4 cm splenomegaly was palpated. Laboratory tests were within normal limits except decreased hematocrit (25%) and red blood cell count (2 250 000/mm$^3$) and a high serum alkaline phosphatase level (12.5 Bessey-Loewry Units). Ultrasonography displayed a cystic lesion 20 cm in

* Correspondence to: Dr. A. Emre, 9–10 Mah A7B 95, 34750 Ataköy, Istanbul, Turkey.

**Table 1** Complications of hydatid cyst in the present series*

| | |
|---|---|
| Total number of patients with liver hydatid cysts (E. Granülosus) | 203 |
| Number of cases with complications | 40 |
| Rupture into the biliary tract | 33 |
| External compression of hydatid cyst onto the biliary passages | 2 |
| Rupture into the peritoneal cavity | 1 |
| Bronchobiliary fistula | 1 |
| Thrombosis of hepatic veins and inferior vena cava | 1 |
| Presinusoidal extrahepatic portal hypertension | 1 |

*Simple suppuration has not been considered as a complication because of its common occurrence.

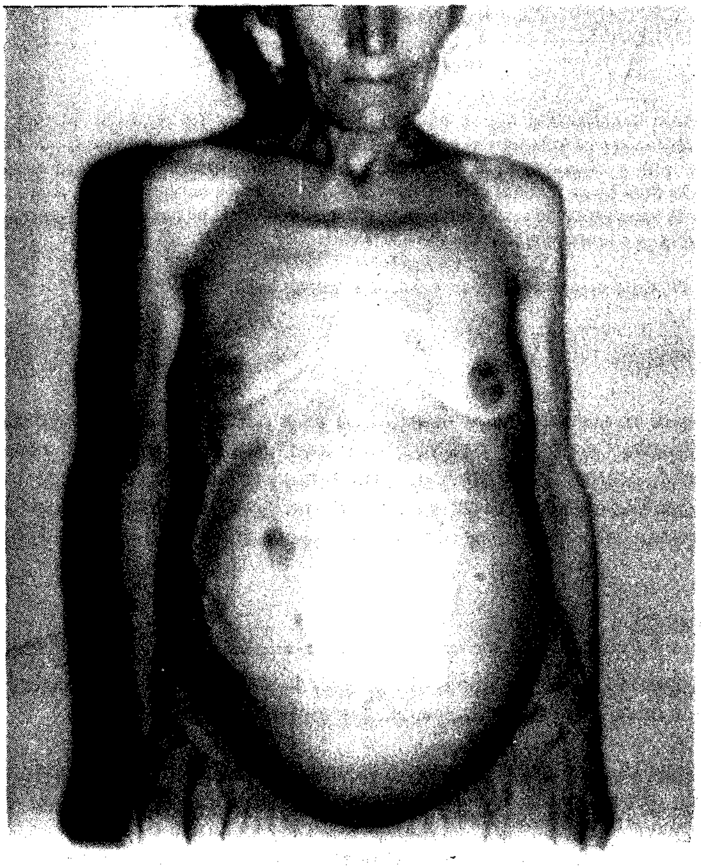

**Figure 1** Ascites and extensive abdominal collaterals of the first case are seen.

diameter within the right lobe of the liver. Oesophageal varices were seen on endoscopic examination. Inferior vena cavogram disclosed thrombosis and widespread collateral circulation above the iliac bifurcation. She was operated on with the diagnosis of extrahepatic presinusoidal portal hypertension due to thrombosis of the hepatic veins and inferior vena cava caused by a liver hydatid cyst. Exploration revealed ascites with diffuse collateral vessel growth in the parietal peritoneum and omentum as signs of portal hypertension, and a hydatid cyst approximately 20 cm in diameter occupying the 5th and 8th segments of the liver. After evacuation of cystic material the operation was completed by obliteration of the cavity with introflecting sutures. Despite the surgical relief of the compression caused by the cyst, ascites reappeared but could be controlled by diuretic therapy. Wedge biopsy of the liver revealed marked congestion and evidence of congestive cirrhosis.

*Case 2*: a 50 year-old male. Four months before his admission, he had a severe gastrointestinal hemorrhage. On physical examination an enlarged liver was palpated 5 cm and 10 cm below the costal arch on the right and left sides respectively. The spleen was enormous in size reaching below the level of the umbilicus. There were no ascites or collaterals. Laboratory investigations revealed moderate pancytopenia. Other tests were within normal limits. Scintigraphy showed a mass in the left lobe markedly displacing the right lobe outwards. Ultrasonography confirmed this finding demonstrating a cystic lesion of the liver. Laparoscopy disclosed a mass covered with omentum. Diffuse oesophageal and fundal varices were observed on endoscopic examination. Transplenic portography displayed a splenic vein thrombosis (Figure 2). At surgery, a hydatid cyst 18 cm in diameter located in the left lobe of the liver and a huge spleen were found. Owing to the extensive collateral growth in the peritoneal cavity and particularly around the cyst, only external drainage of the cyst could be done. Then a splenectomy was performed as portal hypertension was caused by splenic vein thrombosis. He had an uneventful recovery. Endoscopic examination at the 6th month after the operation revealed minimal varices in the distal oesophagus. He is doing well now without any major complaints.

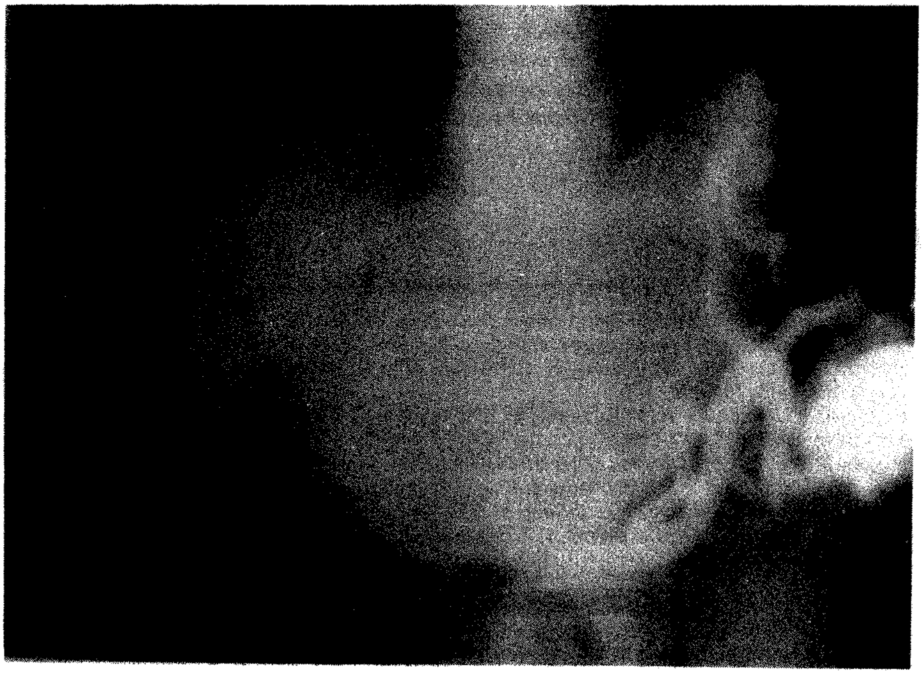

**Figure 2** Transsplenic portogram of the second case.

### DISCUSSION

Hydatidosis is a benign disease. But it may lead to serious complications. These complications are frequently encountered in Turkey where the disease is endemic. Particularly rupture into the biliary passages have been discussed comprehensively in the World Literature[1,2,3,4]. On the other hand, external compression of the hepatic outflow tract by the hydatid cyst and occurence of symptoms are very rare events. Only three cases of hydatid cyst were reported in a large series of Budd-Chiari Syndrome[5]. There are few reports about hydatid cysts causing portal hypertension in the literature as single case reports and these have been diagnosed on postmortem examination[6,7].

One important feature of the first case is cirrhotic transformation subsequent to the diffuse caval and hepatic vein obstruction resulting from the chronic nature of the disease. In such a case, complete relief of symptoms is not expected. However, compression was relieved by this treatment and ascites was partially controlled. The other case is a typical example of gastric portal hypertension secondary to splenic vein thrombosis caused by external compression by the cyst. In this case, splenectomy was performed in addition to hydatid cyst treatment and the patient has benefited from the operation.

A less frequent cause of hydatidosis, Ecchinococcus Alveolaris may result in complications such as extrahepatic portal hypertension, Budd-Chiari Syndrome and extrahepatic cholestasis more often than Ecchinococcus Granulosus. The infiltrative and tumor-like nature of Ecchinococcus Alveolaris lesions is the chief cause of these complications[8,9,10]. During the same period, 10 cases of alveolar ecchinococcosis were encountered in our unit. In 6 of them, extrahepatic cholestasis due to compression or invasion of biliary passages was the main feature (unpublished data).

The principal cause of development of portal hypertension during the course of hydatidosis is the delay of admission to hospital. Although the generally accepted conception in the management of relatively small and intraparenchymal cysts is to follow them up by repeated ultrasonographic examinations, the possible occurrence of complications such as those mentioned here should be kept in mind even though they are not common[11].

### *References*

1. Alper, A., Arioğul, O., Emre, A., Uras, A., Ökten, A. (1987) Choledochoduodenostomy for intrabiliary rupture of hydatid cysts of liver. *Br. J. Surg.* **74**, 243–245.
2. Talib, H. (1968) Some surgical aspects of hydatid disease in Iraq. *Br. J. Surg.* **55**, 576–585.
3. Dadoukis, J., Gamvros, O., Aletras, H. (1984) Intrabiliary rupture of the hydatid cysts of the liver. *World J. Surg.* **8**, 788–790.
4. Ovnat, A., Peiser, J., Avionah, E., Barki, Y., Charuzi, I. (1984) Acute cholangitis caused by ruptured hydatid cyst. *Surg* **95**, 497–500.
5. Parker, R.G.F. (1959) Occlusion of hepatic veins in man. *Medicine* **38**, 369–372.
6. Cox, J.S.T., Seymour, A.E., Clarkson, A.E. (1966) Hydatid disease of the liver associated with the Budd-Chiari Syndrome. *Aust NZ. J. Surg.* **35**, 291–294.
7. Koshy, A., Bushnurmath, S.R., Mitra, K.S., Mahajan, K.K., Datta, D.V., Aikat, B.K., Bhagwat, A.G. (1980) Hydatid disease associated with hepatic outflow tract obstruction. *Am. J. Gastroenterology* **74**, 274–278.
8. Kasai, Y., Koshino, N., Sakamoto, H., Sasaki, E., Komagai, M. (1980) Alveolar ecchinococcosis of the liver: studies on 60 operated cases. *Ann Surg* **191**, 145–152.
9. Khuroo, M.S. Datta, D.U., Khosky, A., Mitra, S.K. (1980) Alveolar hydatid disease of the liver with Budd-Chiari Syndrome. *Postgraduate Med J* **56**, 197–201.

10. Samuels, S., Fosmoe, R. (1970) Alveolar hydatid disease with involvement of the inferior vena cava. *Am J Surg* **36**, 698–701.
11. Little, J.M., Deane, S.A. (1986) Hydatid disease. In Liver Surgery, edited by S. Bengmark, L. Blumgart, pp. 118–129. Edinburgh, London, Melbourne, New York: Churchill Livingstone.

*(Accepted by S. Bengmark 11 April 1989)*

### INVITED COMMENTARY

Emre and colleagues report two cases of an unusual complication of hydatid disease of the liver. The first patient illustrates the problem of hepatic outflow occlusion due to recurrent hydatid disease after two previous operations. Recurrence rates of 5%–10% have previously been reported based mainly on clinical evaluation. A recent careful prospective study using 6-monthly ultrasound and CT scanning in 32 patients identified recurrent hydatid disease in 22% of patients after 30 months.[1] The single major determinant of recurrence by multivariate analysis was previous pre-operative cyst rupture. Careful serial imaging, for at least three years, after surgery is therefore essential in view of recurrent disease occurring in up to a quarter of cases. Many authorities today would advise peri-operative antihelminthic therapy in complex hydatid surgery. Although albendazole achieves substantially higher serum and intracystic levels than mebendazole, neutropenia and toxicity (especially liver and kidney) are potential complications. The optimal dosage and duration of therapy are currently unresolved.

While evacuation of the cyst producing caval and hepatic vein occlusion is the appropriate therapy (Case 1), the persistence of ascites is of concern. Venographic confirmation of restored caval and hepatic vein patency after surgery is important. Persistence of hepatic vein occlusion despite cyst evacuation requires portasystemic shunting or alternatively meso-atrial grafting when associated with inferior vena cava obstruction. While cirrhosis is progressive without adequate outflow decompression, effective shunting has resulted in the documented return to normal of both liver architecture and histology.[2] Substantial series with prolonged survival following liver transplantation for Budd-Chiari Syndrome are now reported.

Jej Krige
Univ. of Cape Town
Cape Town S.A.

